# Natural History of Nonoperatively Treated Borderline Acetabular Dysplasia in Young Adults and Factors Associated With Inferior Functional Outcome

**DOI:** 10.1177/23259671251410199

**Published:** 2026-02-17

**Authors:** Dimitris Dimitriou, Roy Marcus, Dominik Kaiser, Armando Hoch, Patrick Zingg

**Affiliations:** † Department of Orthopedics, University Hospital Balgrist, University of Zurich, Zurich, Switzerland; ‡ Department of Radiology, University Hospital Balgrist, University of Zurich, Zurich, Switzerland; Investigation performed at University Hospital Balgrist, Zurich, Switzerland

**Keywords:** borderline acetabular dysplasia, hip arthroscopy, natural history, periacetabular osteotomy, treatment

## Abstract

**Background:**

The long-term outcomes of nonoperatively treated borderline acetabular dysplasia (BHD) in young adults remain unexplored. This study aimed to investigate the natural history of nonoperatively treated borderline hips over a minimum 10-year follow-up.

**Hypothesis:**

Most patients with nonoperatively treated BHD would exhibit persistent symptoms and radiological evidence of hip osteoarthritis (OA) for at least 10 years.

**Study Design:**

Case series (prognosis); Level of evidence, 4.

**Methods:**

Medical records and radiographs of patients aged 14 to 39 years with BHD, defined as a lateral center-edge angle (LCEA) of 18° to 25°, who presented with hip pain between January 2005 and December 2012, were retrospectively reviewed. Patients treated nonoperatively returned for clinical examination, pelvic radiograph, and hip magnetic resonance imaging (MRI) at a minimum of 10-year follow-up. Medical records, pelvis radiographs, and MR images of patients with borderline hips who underwent surgery, either hip arthroscopy (HAS) for predominantly femoroacetabular impingement (FAI) or periacetabular osteotomy (PAO), were retrospectively reviewed.

**Results:**

Among 45 hips (30 patients) treated nonoperatively (mean age, 23 ± 7 years), 4 hips (9%) showed progression to OA grade 1 at a mean of 15 ± 1 years of follow-up. The mean modified Harris Hip Score (mHHS) was 88 ± 12 at the last follow-up, with only 1 patient scoring <70. Of the surgical group (19 hips, 15 patients), procedures included PAO (5 hips) and HAS (14 hips). Acetabular retroversion (odds ratio [OR], 13 [95% CI, 1.4-122.9]; *P* = .02) and labrum hypertrophy (OR, 17.9 [95% CI, 1.4-228.1]; *P* = .03) correlated with lower mHHS but not with OA progression.

**Conclusion:**

At a mean 15-year follow-up, 19 of 90 (21%) of borderline hips required preservation surgery. Of the 45 nonsurgical hips, 4 (9%) exhibited mild OA progression, and 3 of 4 hips (75%) maintained excellent functional outcomes. Acetabular retroversion and labrum hypertrophy were associated with poorer function but did not predict OA progression. Persistent symptoms were common in patients with labrum hypertrophy after HAS for FAI.

Borderline acetabular dysplasia (BHD) is defined^8^ as a lateral center-edge angle (LCEA) of 18° to 25°. It has a prevalence of 19.8% to 23.3% in the asymptomatic general population and 12.8% in symptomatic patients.^10^ Despite its high prevalence, the optimal treatment approach for patients with symptomatic BHD remains controversial, partly due to the concomitant deformities.^24^ Periacetabular osteotomy (PAO) addresses the structural deformity at the expense of greater surgical exposure,^11,17^ prolonged recovery,^14^ and higher potential complications.^7^ In contrast, arthroscopic capsular plication addresses only the intra-articular pathology in a minimally invasive approach and expedited recovery, without correcting the acetabular bony pathology.^9^ Limited data are available in the literature regarding the nonoperative treatment of borderline hips.

The risk of symptom development and progression of hip osteoarthritis (OA) has been well-established in classic acetabular dysplasia (LCEA<18°).^15,23,33,35^ Thomas et al,^37^ in a 20-year longitudinal cohort of 1003 women, reported that an LCEA <28° was associated with a 13% increased risk of radiographic OA and an 18% risk of total hip arthroplasty (THA), with a linear risk increase for radiographic OA for each degree of LCEA <28. This suggests that patients with BHD might have an increased risk of OA compared with a population with a normal LCEA. Patients with a BHD may develop early OA either due to chronic joint instability leading to chondral surface overload and subsequent articular cartilage and labral damage^21,27^ or due to the commonly associated cam-type morphology (reported^6^ prevalence of about 75%), which might lead to femoroacetabular impingement (FAI), and also a well-established risk factor for hip OA.^2,33^ Despite these observations, the natural history of BHD in young adults has never been investigated.

Therefore, the present study aimed to report the natural history, that is, the development of symptoms requiring surgical intervention and radiological progression of hip OA, as well as the implantation of THA in patients with BHD with a minimum 10-year follow-up. The study hypothesis was that patients with nonoperatively treated BHD would develop symptoms and radiological signs of hip OA.

## Methods

### Study Design, Inclusion and Exclusion Criteria

The present study was approved by the institutional review board and the ethical committee at our institution. The study was conducted entirely at the authors' institution. The medical records and hip radiographs of all patients aged 14 to 39 years who presented at the outpatient hip clinic from January 2005 to December 2012 were retrospectively reviewed. Patients with an LCEA between 18° and 25° were included. The exclusion criteria included patients <14 years or >39 years at the time of the first consultation; previous surgery on the affected hip; and patients with Legg-Calvé-Perthes disease, slipped capital femoral epiphysis, or secondary hip OA.

Patients who met the above-mentioned criteria were invited to the outpatient clinic for a clinical examination, an anteroposterior (AP) pelvic radiograph, and a hip magnetic resonance imaging (MRI). The minimum follow-up was 10 years. The medical records, pelvis radiographs, and hip MR images of borderline hips that underwent surgery—hip arthroscopy (HAS) or PAO—during the above-mentioned period were retrospectively reviewed. A HAS was performed in patients with predominantly FAI symptoms, such as inguinal pain with hip flexion activities, a positive anterior impingement test in the clinical examination, and a concomitant cam deformity radiographically, whereas a PAO was performed in patients with predominantly hip instability symptoms, such as inguinal or lateral hip pain during weightbearing activities and a positive apprehension test in the clinical examination.

### MRI Examination

MRI scans were acquired with a 3T Siemens Skyra-fit scanner (Siemens). Unilateral MRI was performed for each hip separately with the following sequences: Coronal T2-weighted sequence (repetition time/echo time 4000/83 ms; field-of-view 220 mm; slice thickness 4 mm), sagittal T1-weighted sequence (repetition time/echo time 678/14 ms; field-of-view 180 mm; slice thickness 4 mm), transverse short tau-inversion recovery sequence (repetition time/echo time 5000/62 ms; inversion time 210 ms; field-of-view 180 mm; slice thickness 7 mm), transverse T1-weighted sequence (repetition time/echo time 820/13ms; field-of-view 180 mm; slice thickness 6 mm).

### Radiographic Assessment

The radiological evaluation was performed by a fellowship-trained, board-certified musculoskeletal radiologist (with 8 years of experience, M.R.), blinded to all clinical data. Using an AP pelvic radiograph, the following radiographic parameters were evaluated: The Wiberg LCEA,^22^ Tönnis acetabular index (AI),^4^ femoral head extrusion index,^39^ OA grade according to Tönnis, corrected femoroepiphyseal acetabular roof (FEAR) index,^16^ and the caput collum diaphyseal (CCD) angle.^40^ Acetabular retroversion was determined by the concomitant presence of crossover, posterior wall, and sciatic spine signs on conventional radiographs,^4^ which provided a specificity of 94%, as described by Lerch et al.^20^

On the hip MRI, the following parameters were measured: femoral torsion,^34^ iliocapsularis-to-rectus-femoris (IC/RF) cross-sectional ratio,^13^ labral hypertrophy (defined as a height-to-length ratio of <0.5 on coronal MR images) as described by Toft et al,^38^ the presence of cam morphology, herniations pits, labrum lesions, and alpha angle (α°).^28^ The femoral or acetabular cartilage defects were classified as follows: grade 0: intact cartilage, grade 1: ≤50%, grade 2: 50%-75%, and grade 3: >75% defect.

### Clinical Examination and Functional Outcomes

The chart review included patient characteristics (age at first and last presentation, sex, body mass index, occupation, and smoking status), activity level, symptoms, pain location, and pain intensity according to the visual analog scale (VAS). The clinical examination included hip range of motion, limping, anterior impingement, and the apprehension test. The modified Harris Hip Score (mHHS),^31^ Western Ontario and McMaster Universities Arthritis Index (WOMAC),^36^ and subjective hip value^19^ were acquired at the last follow-up.

### Statistical Analysis

Descriptive statistics used means, standard deviations, and ranges to present the data. Patients were divided into the following groups for subgroup analysis: OA progression versus no progression, mHHS >70 versus mHHS <70 (mHHS = 70 was used as a cutoff because mHHS<70 is classified as a poor result), and patients who did not undergo surgery versus PAO versus HAS. All parameters were tested for normality using the Kolmogorov-Smirnov test. When the criteria for normality were met, a 2-tailed *t* test was used; otherwise, the Mann-Whitney *U* test was applied to compare the different parameters between the above-mentioned groups. A univariate logistic regression analysis was performed to evaluate whether OA progression, functional outcomes, or surgery was correlated with patient characteristics and radiological and MR parameters. Only the parameters found to be significant (*P* < .05) in the univariate logistic regression were included in the multiple logistic regression analysis. The level of significance was set at *P* ≤ .05. All statistical analyses were performed using SPSS Version 23 software (IBM).

## Results

### Patient Characteristics

**Figure 1.**
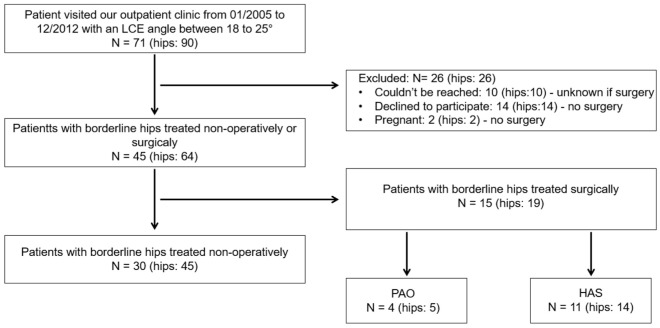
Patient inclusion flow chart. HAS, hip arthroscopy; LCEA, lateral center-edge angle; PAO, periacetabular osteotomy.

A total of 71 patients (90 hips) were identified. Ten patients (14%) could not be reached, 16 (18%) could not participate but reported no surgery on the affected hip, and 15 (19 hips, 21%) underwent surgery (PAO: 5 hips, 6%, HAS: 14 hips, 16%) (Figure 1). No patients underwent THA during the study period. A total of 30 patients (42%) (women: n = 23; men: n = 7; 45 hips) who did not undergo surgery agreed to return to the outpatient clinic for follow-up (Figure 1). The mean age at the first consultation was 23 ± 7 years (range, 14-39 years), and the mean follow-up was 15 ± 4 years (range, 10-26 years) (Table 1).

**Table 1:** Summary of Patient Characteristics, Radiographic and MRI Parameters, and Functional Outcomes^*a*^

	All Patients, N = 45	Hips With no OA Progression, n = 41	Hips With OA Progression, n = 4	Significance, *P*
Characteristics				
Age at first consultation, years	23 ± 7 (14 to 39)	23 ± 7 (14 to 39)	27 ± 4 (21 to 30)	.1
Age at last, years	37 ± 8 (25 to 52)	37 ± 8 (25 to 52)	44 ± 3 (39 to 46)	**<.01**
Female sex	34 (76)	32 (78)	2 (50)	.2
Left side	19 (42)	18 (44)	1 (25)	.5
BMI, kg/m^2^	24 ± 4 (19 to 33)	24 ± 3 (19 to 33)	28 ± 5 (22 to 32)	.3
Sedentary occupation	39 (87)	39 (95)	0	**<.01**
Follow-up, years	15 ± 4 (10 to 26)	14 ± 4 (10 to 26)	15 ± 1 (14 to 16)	.5
Radiographic parameters				
LCEA, deg	21 ± 2 (18 to 25)	21 ± 2 (18 to 25)	22 ± 2 (19 to 24)	.8
AI, deg	10 ± 4 (2 to 18)	10 ± 4 (2 to 18)	8 ± 5 (2 to 15)	.6
Femoral extrusion index	21 ± 4 (6 to 29)	21 ± 4 (6 to 29)	23 ± 5 (17 to 28)	.6
Acetabular retroversion	7 (16)	6 (25)	1 (25)	1
FEAR index, deg	–5 ± 8 (–14 to 10)	–5 ± 8 (–14 to 10)	–9 ± 4 (–12 to −4)	.1
CCD, deg	132 ± 5 (120 to 141)	132 ± 5 (123 to 141)	125 ± 3 (120 to 128)	**<.01**
MRI parameters				
Femoral torsion, deg	22 ± 10 (1 to 45)	22 ± 10 (1 to 45)	20 ± 10 (7 to 31)	.7
Cam morphology	11 (24)	10 (24)	1 (25)	1
A-angle, deg	44 ± 13 (16 to 64)	43 ± 13 (16 to 63)	52 ± 11 (38 to 64)	.2
Labrum hypertrophy	24 (53)	21 (51)	3 (75)	.4
IC/RF cross-sectional ratio	0.93 ± 0.5 (0.23 to 2.2)	0.95 ± 0.5 (0.23 to 2.2)	0.87 ± 0.4 (0.6 to 1.4)	.5
Functional outcomes				
mHHS	88 ± 12 (48 to 100)	87 ± 13 (48 to 100)	86 ± 25 (48 to 100)	.9
WOMAC	1.6 ± 2.2 (0 to 8.7)	1.7 ± 2.3 (0 to 8.7)	2.7 ± 4 (0.3 to 8.7)	.7
SHV	59 ± 36 (20 to 100)	63 ± 33 (20 to 100)	64 ± 32 (20 to 95)	.6

a Data are presented as mean ± SD (range) or n (%). Bold *P* values indicate statistically significant differences (*P* < .05). AI, acetabular index; BMI, body mass index; CCD, caput collum diaphyseal; FEAR, femoroepiphyseal acetabular roof; IC/RF, iliocapsularis-to-rectus-femoris; LCEA, lateral center-edge angle; mHHS, modified Harris Hip Score; MRI, magnetic resonance imaging; OA, osteoarthritis; SHV, subjective hip value; WOMAC, Western Ontario and McMaster Universities Arthritis Index.

### Radiographic Parameters

The mean LCEA was 21° ± 2° (range, 18° to 25°), AI was 10° ± 4° (range, 2° to 18°), and the femoral extrusion index was 21% ± 4% (range, 6% to 29%). The mean corrected femoroepiphyseal acetabular roof (FEAR) index was −5° ± 8° (range, −14° to 10°), and the mean CCD angle was 132° ± 5° (range, 120° to 141°). Acetabular retroversion was present in 7 (16%) hips. One patient (2%) had an OA grade of 2 at the initial presentation and did not progress over 14 years. The rest of the hips (n = 44; 98%) had no radiographic signs of hip OA (Tönnis 0), whereas 4 hips (9%) progressed to OA grade 1 at a mean follow-up of 15 ± 1 (range, 14 to 16) years.

### MRI Parameters

The mean femoral torsion was 22° ± 10° (range, 1° to 45°), the IC/RF ratio was 0.93 ± 0.5 (range, 0.23 to 2.2), and the alpha angle was 43° ± 14° (range, 16° to 64°). Labral hypertrophy was present in 24 hips (53%), cam morphology in 11 hips (24%), herniation pits in 4 hips (9%), and labrum lesions in 30 hips (67%). Femoral cartilage defects grade 1 were present in 10 hips (22%), grade 1 acetabular defects in 15 hips (33%), and grade 3 acetabular defects in 4 hips (9%).

### Clinical Examination and Functional Outcomes

The reason they did not undergo surgery was that their symptoms could be managed adequately with nonoperative measures (ie, physical therapy, nonsteroidal anti-inflammatory drugs, and activity modifications) for at least 3 months. Most patients (87%) had a sedentary occupation and reported symptoms during or after sports (such as jogging [30%] and hiking [22%]), during daily activities (such as climbing stairs [11%]), while sitting (16%), or at night (7%). The mean Tegner score at the first and last consultation was 7 ± 2 (range, 2-9) and 6 ± 1 (range, 2-8), respectively. About 40% of patients reported stopping activities such as football, jogging, or other sports because of pain.

Inguinal pain was reported in 44% of patients, with a mean visual analog scale (VAS) of 1.4 ± 2 (range, 0 to 7) points, whereas 53% reported trochanteric pain, with a mean VAS of 2.2 ± 2.7 (range, 0 to 8) points. Finally, 27% of the patients reported no pain at the follow-up.

The mean hip range of motion consisted of flexion 105° ± 10° (range, 90° to 130°), extension 5° ± 5° (range, 0° to 10°), internal rotation at 90° of flexion: 35° ± 10° (range, 10° to 60°), and external rotation at 90° of flexion: 40° ± 10° (range, 20° to 60°). The anterior impingement test was positive in 10 (22%) hips, whereas the apprehension test was positive in 18 (40%) hips.

The mean mHHS and WOMAC scores were 88 ± 12 (range, 48 to 100) and 1.6 ± 2.2 (range, 0 to 8.7), respectively. The mean subjective hip value was 74% ± 20% (range, 20% to 100%).

### Subgroup Analysis (OA Progression Versus No Progression)

Four hips (9%) demonstrated OA progression over a mean of 15 ± 1 years. No significant differences in patient characteristics, radiographic, or MR parameters were found between patients with OA progression and those without progression. Despite the radiographic OA progression, the mean mHHS was 86 ± 25 points, with only 1 patient reporting an mHHS <70. The multivariable logistic regression analysis demonstrated no association between patient characteristics, radiological parameters, and MR parameters with OA progression.

### Subgroup Analysis (mHHS >70 Versus mHHS<70)

Five hips (11%) had an mHHS <70, and only 1 patient with OA progression had an mHHS <70. The multivariable logistic regression analysis demonstrated that the presence of acetabular retroversion (odds ratio [OR], 13 [95% CI, 1.4 to 122.9]; *P* = .02) and labral hypertrophy (OR, 17.9 [95% CI, 1.4 to 228.1]; *P* = .03) were correlated with a lower mHHS.

### Subgroup Analysis (No Surgery Versus HAS Versus PAO)

A total of 15 patients (19/80 hips; 24%) with BHD underwent HAS (n = 14 hips) or PAO (n = 5 hips). The reason they underwent surgery was persistent symptoms despite nonoperative treatment for 3 months (physical therapy, nonsteroidal anti-inflammatory drugs, or activity modifications). Patients who underwent PAO were younger at the first consultation with a mean age of 19 ± 5 years compared with patients who underwent HAS (24 ± 4 years) or no surgery (23 ± 7 years) (*P* < .05). All hips that underwent HAS for FAI had cam deformity and labrum lesions, whereas 2 (14%) had acetabular retroversion and 4 (28%) had labral hypertrophy. Of the 4 patients with labrum hypertrophy treated with a HAS for FAI, 3 (75%) had persistent symptoms. All hips that underwent PAO demonstrated labral hypertrophy, with 2 (40%) having labrum lesions and 2 (40%) having cam morphology. The LCEA, AI, extrusion index, and femoral torsion were similar between groups. The multivariable logistic regression analysis did not demonstrate any correlation between patient characteristics, radiological parameters, MR parameters, and surgical treatment.

## Discussion

The optimal treatment option for BHD remains, partly due to the high incidence of concomitant deformities (especially FAI), largely unknown. Regarding the nonoperative treatment of symptomatic BHD, limited data are available in the literature. The present study aimed to report the natural history of nonoperatively treated BHD in symptomatic young adults. At a mean 15-year follow-up, about one-fourth of the patients with symptomatic borderline hips underwent hip preservation surgery, whereas no patient underwent a THA. Of the patients who did not undergo surgery, 9% demonstrated a slight increase in OA progression (grade 1) on radiograph. Nevertheless, the majority (75%) reported excellent functional outcomes, comparable to those of patients who did not undergo surgery. No correlation was found between demographic characteristics, radiological and MR parameters, and OA progression or hip preservation surgery. However, the presence of labrum hypertrophy and acetabular retroversion was correlated with worse functional outcomes.

Mild or BHD might also increase the risk of radiographic OA development and THA. In the present study, 30 young adults (45 hips) presenting to the outpatient clinic with hip pain and an LCEA between 18° and 25° were assessed clinically and radiographically to evaluate the risk of OA development. Over a mean 15-year follow-up, only 4 hips (9%) demonstrated mild OA progression (Tönnis grade 0 to grade 1), and no patient underwent a THA. Despite OA progression, these patients demonstrated excellent functional outcomes, similar to those observed in patients without OA progression. The difference between the observed data and the population-based data reported by Thomas et al^37^ might be due to age differences between the study populations. In the present study, the mean age at the first and last consultations was 23 and 37 years, respectively, whereas in the study by Thomas et al,^37^ it was 54 years at the beginning of the study and 74 years at the last follow-up. In a cross-sectional study of about 600,000 people aged ≥60 years (mean age, 74.9 years) in Germany, Postler et al^30^ reported that 23.9% of female patients were diagnosed with hip or knee OA. These data may suggest that young adults with symptomatic hip dysplasia who manage their symptoms with nonoperative measures (physical therapy, nonsteroidal anti-inflammatory drugs, or activity modifications) are at low risk of mild radiographic OA progression yet still achieve excellent functional outcomes (mHHS >85%) over a mean 15-year period. Therefore, surgical treatment should be reserved for patients with BHD with persistent symptoms despite nonoperative management for at least 3 months.

Surgical treatment for BHD depends on the stability of the hip and includes nonoperative treatment and surgical interventions to address either intra-articular FAI (HAS) or instability (PAO).^41^ Regarding functional outcomes after treatment of BHD, limited data are available, and the literature has primarily focused on arthroscopy outcomes, with only a few small reports on PAO outcomes. Nawabi et al^25^ reported >20 points of average improvement (preoperative: 61.7 to postoperative: 86.2) in the mHHS after HAS (with labral refixation and capsular closure) in 46 patients (55 hips) with BHD and concomitant FAI, with comparable outcomes to nondysplastic patients. Similarly, Domb et al,^8^ in a retrospective study of 24 patients (25 hips) with BHD treated with labral preservation and capsular plication, reported a mean mHHS increase from 70.3 to 85.9 at 68.8-month follow-up. In a retrospective analysis of 77 patients with BHD by Andronic et al,^1^ a total of 28 patients treated with a PAO and 49 with HAS reported a mean improvement in mHHS of 14.9 and 22.5 points, respectively, with a mean mHHS at the last follow-up (minimum 5 years postoperatively) of 89.4 and 93.4. Clohisy et al^5^ in a prospective, multicenter cohort of 320 dysplastic hips (35% with LCEA >15°) treated with a PAO reported a mean mHHS increase from 58 to 81 in hips with an LCEA >15° at a minimum 2-year follow-up. However, they concluded that male sex and mild acetabular dysplasia were predictive of lesser improvements in certain outcome measures. Similarly, Nepple et al,^26^ in a retrospective study of 186 borderline hips treated with a PAO, reported a mean increase in mHHS from 58 to 86 at 3.3-year follow-up. Regarding nonoperative treatment, the present study is the only one available in the literature. At a mean follow-up of 15 years, the mean mHHS was 88, with only 11% of patients reporting an mHHS of <70. Therefore, the functional outcomes of nonsurgically treated borderline hips were similar to those reported in the literature after HAS and PAO, suggesting that nonoperative treatment might be a reasonable option if symptom relief can be achieved with nonoperative measures.

Acetabular retroversion consists of a malorientation of the acetabulum in the sagittal plane, resulting in poor posterior coverage and excessive anterior marginal prominence.^12^ In the present study, patients with concomitant BHD and acetabular retroversion had a higher probability of lower functional outcomes than patients without acetabular retroversion. In normal hips, the highest region of contact and articular load in the acetabulum is located in the posterosuperior aspect.^29^ In retroverted hips, the decreased area posteriorly modifies the articular contact region, causing nonhomogeneously distributed stress throughout the articular surface, leading to subsequent hip pain and early development of OA.^12,32^ The degenerative mechanisms resulting from anterior hyper-coverage are due to a combination of labral lesions and *contrecoup lesions* on the posterior wall.^2^

A larger acetabular labrum might be associated with acetabular dysplasia and clinical symptoms. Kamenaga et al,^18^ in an attempt to investigate potential associations between labral length, acetabular morphology, and clinical symptoms, retrospectively evaluated 102 patients who presented to their hip joint clinic. Patients with frank acetabular dysplasia (LCEA <18°) had a significantly greater labral length (10.5 ± 2.9 mm) than patients with BHD (8.3 ± 2 mm). Patients with borderline hips and labral lengths >10 mm had significantly higher symptom ratios (10/11; 90.9%) than those with lengths <10 mm (26/53; 49.1%; *P* < .001). In the present study, labrum hypertrophy was correlated with worse mHHS. Labral hypertrophy may indicate a compensatory response to a lack of bony coverage, which may ultimately result in degenerative changes and symptoms. Although labrum hypertrophy was associated with worse functional scores, it was not correlated with OA progression. However, the majority of the patients with BHD and concomitant labrum hypertrophy had persistent symptoms after a HAS for FAI. This finding was also supported by Brinkman et al,^3^ who reported inferior postoperative outcome scores for arthroscopic labral repair in patients with FAI and a hypertrophic labrum, as measured intraoperatively.

### Limitations

The present study should be interpreted in light of its potential limitations. The most obvious challenge was the relatively small sample size, especially for the subgroup analysis. Therefore, we could not identify risk factors that could predict the progression of hip OA. Also, due to the small sample size, the confidence interval for the multivariable regression analysis was extremely wide, suggesting a possible association, but the precision was poor, and more robust data would be needed to confirm the effect size. Furthermore, although the mean follow-up time was 15 years, the mean patient age at final follow-up was 37 years, which might still be early for OA development. Finally, a control group with operatively treated borderline hips was not available for a direct comparison, as the main purpose of the present study was to report the natural history of borderline hips. Instead, the functional outcomes were compared with borderline hips treated with HAS or PAO, as reported in the literature.

## Conclusion

The present study is the only one in the literature to report the natural history of BHD in young adults. At a mean 15-year follow-up, about one-fourth of patients with symptomatic borderline hips underwent hip preservation surgery, whereas no patients underwent total hip replacement. Although 4 of 45 (9%) borderline hips that did not undergo surgery showed a slight increase in OA progression on radiographs, the majority (3/4, 75%) reported excellent functional outcomes, similar to those who did undergo surgery. These results suggest that nonoperative treatment of BHD might be a reasonable option if symptom relief can be achieved with nonoperative measures. The presence of labral hypertrophy and acetabular retroversion was associated with inferior functional outcomes; however, neither was associated with OA progression. The majority of the patients with BHD and concomitant labrum hypertrophy had persistent symptoms after a HAS for FAI.
